# *Ureaplasma parvum* meningitis following atypical choroid plexus papilloma resection in an adult patient: a case report and literature review

**DOI:** 10.1186/s12879-021-06975-y

**Published:** 2021-12-20

**Authors:** Na Xing, Zhenxiang Zhao, Qingjing Li, Yalan Dong, Jianfeng Li, Shuping Zhang

**Affiliations:** 1 Department of Endocrinology, The Fourth Hospital of Hebei Medical University, No. 12, Jiankang Road, Shijiazhuang, 050000 Hebei People’s Republic of China; 2 Department of Neurosurgery, The Fourth Hospital of Hebei Medical University, No. 12, Jiankang Road, Shijiazhuang, 050000 Hebei People’s Republic of China; 3 Clinical Laboratory, The Fourth Hospital of Hebei Medical University, No. 12, Jiankang Road, Shijiazhuang, 050000 Hebei People’s Republic of China; 4 Department of Oncology, The Seventh People’s Hospital of Hebei Province, No. 389, Jungong Road, Dingzhou, 073000 Hebei People’s Republic of China

**Keywords:** *Ureaplasma parvum*, Meningitis, Adult, Case report, Metagenomic next-generation sequencing (mNGS)

## Abstract

**Background:**

While *Ureaplasma parvum* has previously been linked to the incidence of chorioamnionitis, abortion, premature birth, and perinatal complications, there have only been rare reports of invasive infections of the central nervous system (CNS) in adults. Owing to its atypical presentation and the fact that it will yield sterile cultures using conventional techniques, diagnosing *U. parvum* meningitis can be challenging.

**Case presentation:**

We describe a case of *U. parvum* meningitis detected in an adult patient following surgical brain tumor ablation. After operation, the patient experienced epilepsy, meningeal irritation, and fever with unconsciousness. Cerebrospinal fluid (CSF) analysis showed leukocytosis (484 * 10^6^ /L), elevated protein levels (1.92 g/L), and decreased glucose concentrations (0.02 mmol/L). Evidence suggested that the patient was suffering from bacterial meningitis. However, no bacterial pathogens in either CSF or blood were detected by routine culture or serology. The symptoms did not improve with empirical antibiotics. Therefore, we performed metagenomic next-generation sequencing (mNGS) to identify the etiology of the meningitis. *Ureaplasma parvum* was detected by mNGS in CSF samples. To the best of our knowledge, this case is the first reported instance of *U. parvum* meningitis in an adult patient in Asian. After diagnosis, the patient underwent successful moxifloxacin treatment and recovered without complications.

**Conclusions:**

As mNGS strategies can enable the simultaneous detection of a diverse array of microbes in a single analysis, they may represent a valuable means of diagnosing the pathogens responsible for CNS infections and other clinical conditions with atypical presentations.

## Background

*Ureaplasma* species, which include the human associated *Ureaplasma parvum* and *Ureaplasma urealyticum*, are common commensal microorganisms found within the genitourinary tract which lack cell walls and are among the smallest known free-living organisms [1]. These microbes are associated with a range of obstetrical complications, including miscarriage, chorioamnionitis, stillbirth, premature birth, and neonatal meningitis [1, 2]. These microbes can also give rise to urogenital infections in adults [3]. In rare cases, *U. parvum* can cause a form of meningitis that is often difficult to diagnose owing to its atypical presentation and the abnormal characteristics of these bacteria. Specifically, *U. parvum* cannot survive under traditional culture conditions and cannot be detected by Gram staining owing to their small size and lack of a cell wall [4]. In contrast with these traditional techniques, metagenomic next -generation sequencing (mNGS) screening is an approach that can be used to detect unexpected or novel pathogens associated with infections of the central nervous system (CNS) [5]. As *U. parvum* infections are rare outside of the urogenital tract in adults [6, 7], the use of such alternative diagnostic techniques may be invaluable in affected individuals. Herein, we describe a rare case of *U. parvum* meningitis that was ultimately diagnosed via mNGS in an adult patient that had undergone atypical choroid plexus papilloma resection.

## Case presentation

**Fig. 1:**
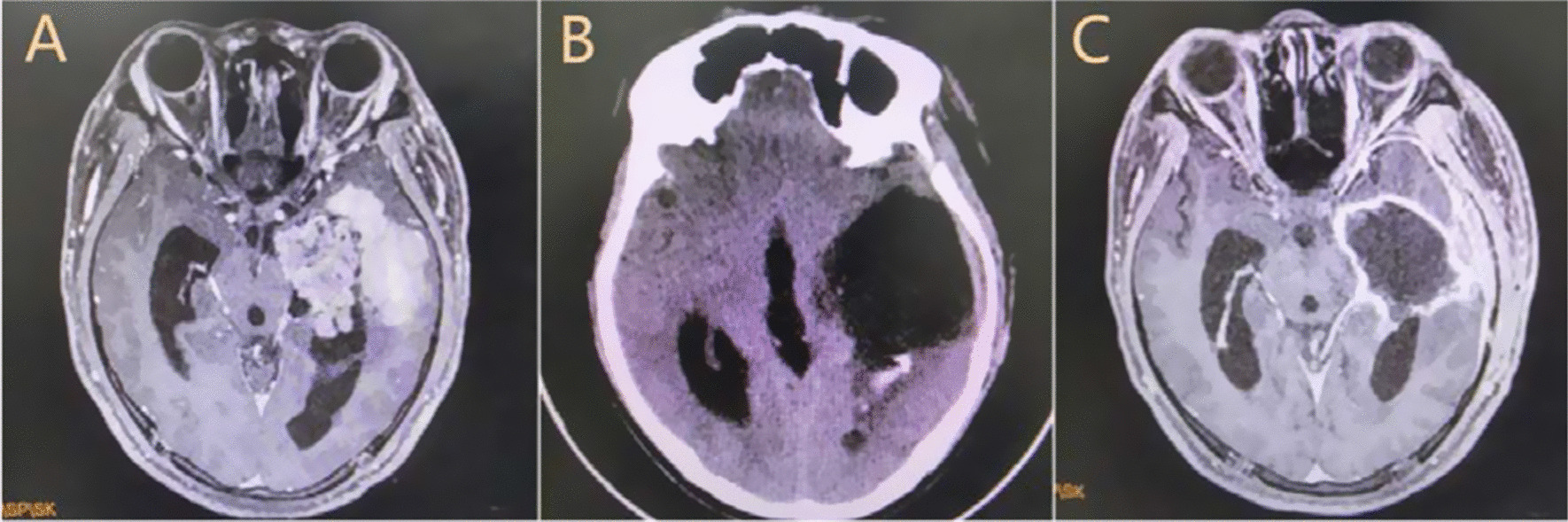
**A** Enhanced preoperative MRI showing a left temporal lobe-lateral ventricle tumor. **B** CT scans on the first day after surgery demonstrating that the tumor had been removed and replaced with CSF, with the opening of the left lateral ventricles. **C** Enhanced MRI images on the 43rd day after surgery demonstrating complete tumor resection

**Fig. 2:**
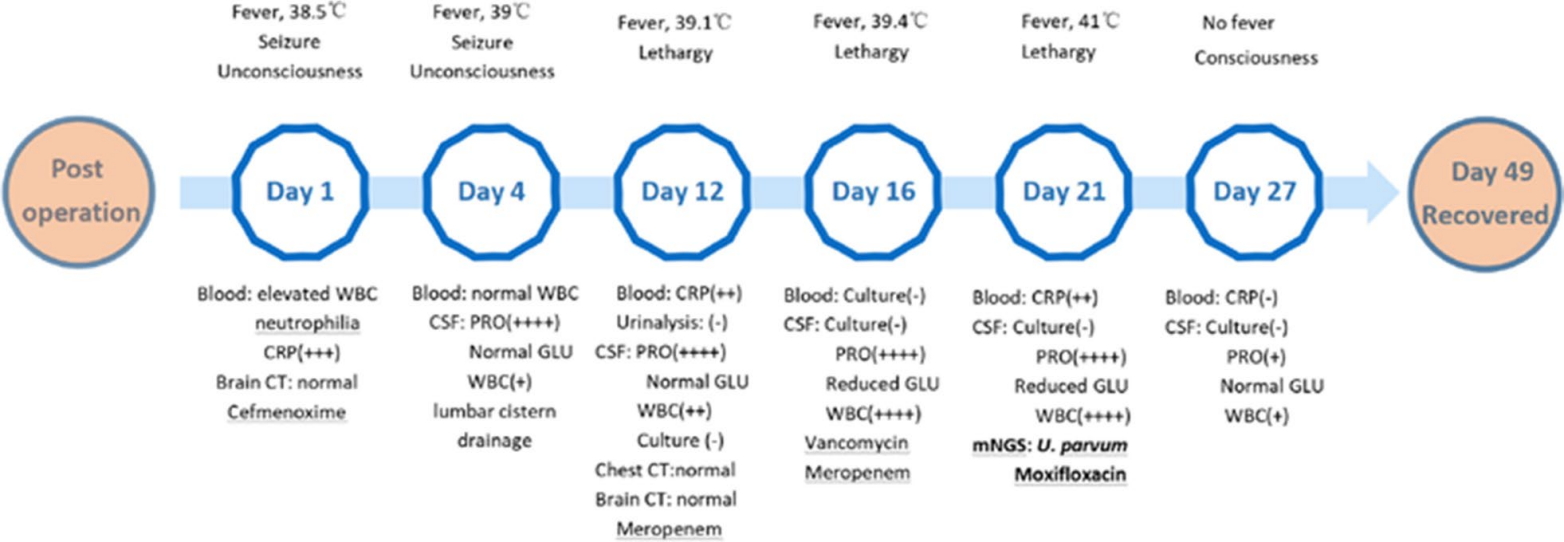
The timeline of the clinical presentation, testing, and treatment for the present case. *WBC* white blood cell, *PRO* protein, *GLU* glucose

A 22-year-old male was admitted to our hospital to undergo the surgical resection of a lateral ventricle tumor affecting the left temporal lobe (Fig. 1A). His medical history did not exhibit any other specific or noteworthy findings. On the first day after surgery, the patient experienced epilepsy, meningeal irritation, and fever with unconsciousness (38.5 °C). Cerebral computed tomography (CT) scans did not detect any bleeding or cerebral edema, and no significant ventricle dilation was evident as compared to preoperative images (Fig. 1B). Blood analyses revealed evidence of leukocytosis (15.53 * 10^9^/L) and elevated C-reactive protein (CRP) levels (168 mg/L), while procalcitonin (PCT) levels were within the normal range (0.67 ng/mL). In light of the possibility that the patient was suffering from an intracranial infection, he was administered cefmenoxime (2 g IV per 12 h). As a semisynthetic third-generation cephalosporin, cefmenoxime exhibits potent activity against a wide variety of pathogens including *Neisseria meningitidis*, *Haemophilus influenzae*, *Streptococcus pneumoniae*, and most gram-negative bacilli [8]. However, we were unable to rule out the possibility of aseptic inflammation due to the opening of the ventricle (Fig. 1B). As such, efforts were made to culture microorganisms from blood and cerebrospinal fluid (CSF) samples. Following antibiotic treatment for two days, the patient’s white blood cells counts had returned to normal but his fever had become more severe (39 °C). CSF analyses revealed leukocytosis (18 * 10^6^/L), elevated protein levels (1.5 g/L), and normal glucose levels (3.24 mmol/L). To replace the CSF and alleviate meningeal irritation, lumbar cistern drainage was conducted following the removal of the cerebral drainage tube. Following a 7-day observation period, no microorganisms were detected in cultures or Gram staining assays prepared from blood and CSF samples. On day 13 after surgery, no improvements in fever or unconsciousness were observed for this patient. While his CRP levels (97.6 mg/L) were substantially lower than at earlier time points, they nonetheless remained high above normal levels (< 6 mg/L). When CSF analyses were repeated at this time point, they indicated leukocytosis (484 * 10^6^ /L), elevated protein levels (1.92 g/L), and decreased glucose concentrations (0.02 mmol/L). As such, growing evidence suggested that the patient was suffering from bacterial meningitis. Antibiotic treatment protocols were thus modified to consist of vancomycin (1 g IV per 12 h) and meropenem (1 g IV per 8 h). However, bacteriological cultures of blood and CSF remained sterile. While the patient regained consciousness, his fever did not abate. Given that efforts to culture CSF samples had failed, additional CSF samples were collected and sent for unbiased mNGS analysis (Vision Medicals, Guangzhou, China). Within 72 h, this approach yielded 16.16 million sequences and detected 8267 sequences consistent with *U. parvum* (Tables 1, 2). As such, the patient was tentatively diagnosed with *U. parvum* meningitis and was treated with moxifloxacin (0.4 g IV per 24 h) while being weaned of other antibiotics. Following a 2-day moxifloxacin treatment period, his fever subsided and his condition gradually improved. After the completion of a 2-week moxifloxacin course, his body temperature remained within normal ranges and he was discharged. The patient was instructed to take oral moxifloxacin for 2 weeks after discharge (Fig. 2). As of writing, it had been 17 months since the patient underwent surgery, and he continued to exhibit good prognosis.

**Table 1:** mNGS result of CSF in this case

Genus			Species		
Generic name	Relative abundance (%)	Number of sequences	Species name	Identification credibility (%)	Number of sequences
*Ureaplasma*	98.7	11,409	*Ureaplasma parvum*	99.0	8267

**Table 2:**
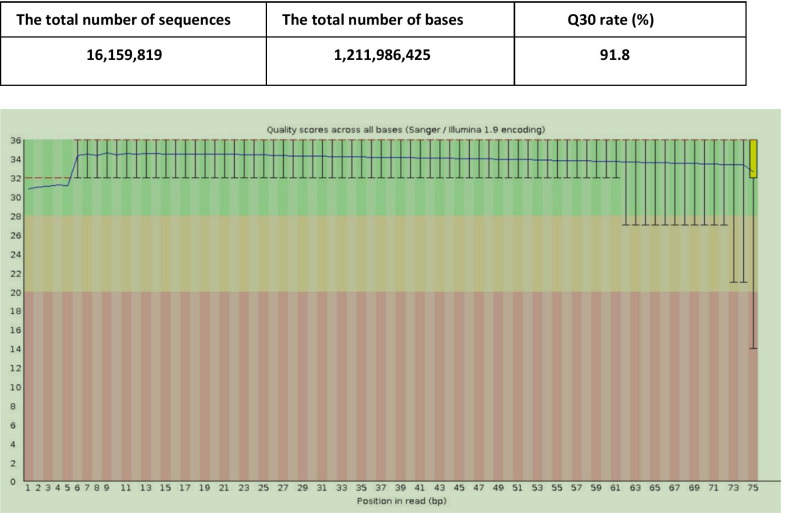
Quality control report of mNGS in this case

## Discussion and conclusions

Herein, we describe a rare case of *U. parvum* meningitis in an adult patient following atypical choroid plexus papilloma resection. While traditional blood and CSF cultures were negative from this patient, a positive pathogen diagnosis was ultimately made via mNGS and the patient underwent successful moxifloxacin treatment.

*Ureaplasma* species are most commonly regarded as pathogens of the genitourinary system wherein they are associated with histological chorioamnionitis, abortion, stillbirth, premature birth, and perinatal complications [1, 2]. The vertical or in utero transmission of *Ureaplasma* species can also result in *Ureaplasma* meningitis in newborns [1], but such infections remain rare among adults. However, there have been other prior reports of postoperative extragenital infections to date (Table 3). For example, in one case a male who had undergone superior third ascending aorta replacement developed mediastinitis, pleuritis, and pericarditis as a consequence of *U. urealyticum* and *Mycoplasma hominis* infection [9]. In another case, a patient that had undergone coronary artery bypass grafting developed a subsequent sternal wound infection [10], while a separate patient was diagnosed with a *U. urealyticum* sternal wound infection following bioprosthetic aortic valve replacement [11]. In 2008, a 38-year-old patient who had undergone complicated kidney transplantation and organ rejection was the first reported adult to be diagnosed with *U. urealyticum* meningitis [12]. In that case, the authors speculated that hematogenous spread was responsible for this case of *Ureaplasma* meningitis. Only one previous report of *U. parvum* meningitis in an adult has been published to date, to the best of our knowledge. Specifically, in 2019, Pailhoriès et al. reported on a case of meningitis that developed in an immunocompetent adult 17 days following surgical craniopharyngioma ablation. No microbes were detected through traditional culture techniques, but *U. parvum* was detected via 16S rDNA sequencing in the CSF. These authors again speculated that *Ureaplasma* hematogenous spread after urinary catheterization was the likely cause of this infection [13]. In the present case, the patient remained catheterized for over two weeks owing to his prolonged unconsciousness, which may have increased his susceptibility to infection. Unfortunately, owing to the high associated costs, we did not conduct mNGS testing of his blood or urine. In addition, prolonged surgery (8 h) may have increased the chance of *U. parvum* infection [14].

**Table 3:** Characteristics of nine adult patients with non uro-genital post-operative *Ureaplasma* infections

Year	Age, sex	Underlying conditions	Surgical method	Clinical features	Specimen	Species	Detection method	Treatment	Refs.
2020	22, M	Atypical choroid plexus papilloma	Resection of atypical choroid plexus papilloma	Meningitis	CSF	*U. parvum*	mNGS	Moxifloxacin	This study
2018	29, M	Craniopharyngioma	Ablation of craniopharyngioma	Meningitis	CSF	*U. parvum*	16S rDNA Sequencing and PCR	Levofloxacin	[13]
2017	31, F	Polymyositis	Lung transplantation	Sepsis	Bronchoalveolar lavage fluid	*U. parvum*	PCR and culture	Azithromycin Doxycycline	[6]
2013	20, M	Burkitt lymphoma	Mastoidectomy	Brain abscess	Abscess fluid	*U. urealyticum*	16S rDNA sequencing and culture	Doxycycline Clarithromycin	[28]
2010	41, M	Endocarditis	Aortic valve replacement	Sternal wound infection	Wound sample	*U. urealyticum*	16S rDNA sequencing	Clarithromycin	[11]
2009	66, M	Cardiovascular disease hypertension diabetes	Three-vessel coronary artery by-pass grafting	Sternal wound infection	Wound sample	*U. parvum*	PCR	Ciprofloxacin	[10]
2008	66, M	Hypertension COPD	Aortic valve and superior third ascending aorta replacement	Mediastinitis pleuritic pericarditis	Wound sample pleural fluid pericardial fluid	*U. urealyticum*	Culture and 16S rDNA sequencing	Doxycycline Clindamycin	[9]
2007	54, M	Non Hodgkin Lymphoma	Total hip prosthesis implantation and resection of abdominal aortic aneurysm	Arthritis and disseminated infection	Aorta tissue and hip soft tissue	*U. parvum*	Real-time PCR	Moxifloxacin	[7]
1995	63, M	Cardiovascular disease hypertension diabetes	Coronary bypass	Sternal wound infection	Wound sample	*U. urealyticum*	Culture	Clindamycin Gentamicin Doxycycline	[29]

*CSF* cerebrospinal fluid, *COPD* chronic obstructive pulmonary disease

The CSF characteristics associated with *U. parvum* meningitis may be varied and atypical. Indeed, in the present case, within two weeks of fever onset, the glucose levels in the CSF did not decrease, and microbial cultures were negative such that we initially suspected a case of aseptic inflammation or viral infection. However, after 16 days of fever, CSF samples exhibited characteristics consistent with bacterial meningitis including leukocytosis, elevated protein levels, and reduced glucose levels. We therefore enhanced the antibiotic grade, but this failed to alleviate their condition. *Ureaplasma parvum* was ultimately only detected via an mNGS approach. Given that *Ureaplasma* species lack a cell wall, antibiotics that disrupt DNA or protein synthesis may be more effective in therapeutic contexts, including macrolides, tetracyclines, chloramphenicol, and quinolones [15]. However, the most appropriate duration of antibiotic therapy for *Ureaplasma* meningitis remains to be defined. In this case, the patient recovered following a 4-week moxifloxacin treatment course.

In one recent report, researchers utilized human brain microvascular endothelial cells (HBMECs) to model *Ureaplasma* meningitis. In this system, the authors found *Ureaplasma* species to be highly pro-inflammatory in these cells, promoting the upregulation of atypical chemokine receptor 3 (AKCR3). The simultaneous inhibition of inflammatory cell death in this context may further disrupt host defense strategies [16]. HBMECs are also primary components of the blood–brain barrier, and by promoting their apoptotic death, *Ureaplasma* species may compromise this barrier and thus increase the susceptibility of the CNS to secondary injury [17]. These effects may also be enhanced in the context of co-infections, consistent with the potential immunomodulatory effects of *Ureaplasma* [18]. This research revealed (at least partly) the pathophysiological mechanism of *Ureaplasma* meningitis. Furthermore, other researchers have found that *Ureaplasma* can modulate host innate immune responses by promoting the downregulation of defense genes in cells, disrupting host membranes using phospholipase, and inducing cytotoxicity via ammonia accumulation or pH-shifts mediated by the hydrolysis of urea [19, 20].

As mentioned above, *Ureaplasma* species fail to survive in standard media, PCR assays designed to test for a wide range of bacterial species represent another approach to *Ureaplasma* detection that is superior to traditional culture techniques. However, these tests are generally hypothesis-driven and thus have the potential to overlook microbes that are not considered to be relevant. Although PCR tests targeting the conserved 16S ribosomal RNA gene (16S PCR) regions of bacteria have been developed, concerns have been raised regarding detection sensitivity. In contrast, as it is based on the Illumina high-throughput sequencing platform, mNGS enables the simultaneous detection of nearly all known pathogens within clinical samples. Recently, Gu et al. presented a case series evaluating the performance of such mNGS testing, highlighting the potential utility of this approach via the detection of pathogens in 7 of 12 (58.3%) cases for which culture and 16 s PCR testing were negative, with subthreshold detection of pathogen reads in an additional two cases (9/12, 75%). They noted that false-negative 16S PCR results are generally attributed to suboptimal primer design or decreased sensitivity from background contamination [21]. In 2008, Palacios et al. employed unbiased metagenomics to detect a novel arenavirus in samples from three patients that had all been implanted with organs from a single donor [22]. They were subsequently able to validate their findings through a combination of PCR, culture, immunohistochemistry, and serological analyses. That study was the first to demonstrate the value of mNGS as an approach to identifying causal pathogens associated with chronic or acute infections. In the years since the publication of that initial report, mNGS-based approaches have been applied with increasing frequency in clinical contexts, with several reports having demonstrated the successful application of mNGS to analyze dozens of sample types including blood, urine, tissue samples, feces, respiratory secretions, and CSF [23]. In one large cohort study of 511 clinical samples, researchers found mNGS to exhibit a detection sensitivity higher than that of culture-based techniques (50.7% vs. 35.2%) while being less significantly impacted by previous exposure to antibiotics [24]. Relative to traditional approaches, mNGS has also been found to detect clinically relevant microorganisms in a larger percentage of immunocompromised individuals [25]. Most mNGS platforms utilized at present can analyze samples within an average of 48 h (range: 24–72 h), and third-generation sequencing technologies can further reduce these times to just a few hours [26]. As such, there is growing clinical recognition of the value of mNGS as a last resort tool for clarifying the causative pathogens in infected patients [23]. In general, mNGS tests are considered only when patients have been hospitalised for extended periods and subjected to costly and invasive testing [27]. Similarly, mNGS was not immediately implemented when this patient developed an intracranial infection. Instead, after multiple failed efforts to culture pathogens from this patient’s blood and CSF samples, an mNGS analysis approach was employed. As sequencing technologies continue to advance and clinicians become more adept in leveraging these tools, we predict that mNGS testing will become an increasingly routine clinical technique.

In summary, this case highlights the importance of considering urogenital commensals as potential causes of postoperative infections of the CNS and exploring the consequences of such infections in detail. The detection of *U. parvum* through traditional techniques is challenging, potentially enabling it to evade detection and thus delaying patient diagnosis and treatment. Both the presenting symptoms and the CSF changes associated with *Ureaplasma* infections may be atypical. As such, any patients with suspected CNS infections without an obvious cause should undergo timely mNGS testing, as the early diagnosis and appropriate treatment of infected individuals may aid in the prevention of adverse outcomes.

## Data Availability

The datasets used and/or analysed during the current study are available from the corresponding author on reasonable request.

## Abbreviations

*U.** Parvum*: *Ureaplasma parvum*
*U.** urealyticum*: *Ureaplasma urealyticum*
CSF: Cerebrospinal fluid
CNS: Central nervous system
mNGS: Metagenomic next-generation sequencing
PCT: Procalcitonin
CRP: C-reactive protein
PCR: Polymerase chain reaction
CT: Cerebral computed tomography
AKCR3: Atypical chemokine receptor 3
HBMECs: Human brain microvascular endothelial cells

## References

[1] Silwedel C, Speer CP, Glaser K (2017). Ureaplasma-associated prenatal, perinatal, and neonatal morbidities. Expert Rev Clin Immunol.

[2] Oh KJ, Romero R, Park JY (2019). The earlier the gestational age, the greater the intensity of the intraamniotic inflammatory response in women with preterm premature rupture of membranes and amniotic fluid infection by *Ureaplasma* species. J Perinat Med.

[3] Beeton ML, Payne MS, Jones L (2019). The role of *Ureaplasma* spp. in the development of Nongonococcal urethritis and infertility among men. Clin Microbiol Rev.

[4] Waites KB, Katz B, Schelonka RL (2005). Mycoplasmas and Ureaplasma as neonatal pathogens. Clin Microbiol Rev.

[5] Gu W, Miller S, Chiu CY (2019). Clinical metagenomic nextgeneration sequencing for pathogen detection. Annu Rev Pathol.

[6] Fernandez R, Ratliff A, Crabb D, Waites KB, Bharat A (2017). Ureaplasma transmitted from donor lungs is pathogenic after lung transplantation. Ann Thorac Surg.

[7] MacKenzie CR, Nischik N, Kram R, Krauspe R, Jäger M, Henrich B (2010). Fatal outcome of a disseminated dual infection with drug-resistant *Mycoplasma hominis* and *Ureaplasma parvum* originating from a septic arthritis in an immunocompromised patient. Int J Infect Dis.

[8] Humbert G, Veyssier P, Fourtillan JB (1986). Penetration of cefmenoxime into cerebrospinal fluid of patients with bacterial meningitis. J Antimicrob Chemother.

[9] García-de-la-Fuente C, Miñambres E, Ugalde E, Sáez A, Martinez-Martinez L, Fariñas MC (2008). Post-operative mediastinitis, pleuritis and pericarditis due to *Mycoplasmahominis* and *Ureaplasma urealyticum* with a fatal outcome. J Med Microbiol.

[10] Walkty A, Lo E, Manickam K, Alfa M, Xiao L, Waites K (2009). *Ureaplasma parvum* as a cause of sternal wound infection. J Clin Microbiol.

[11] Lucke K, Kuster SP, Bertea M, Ruef C, Bloemberg GV (2010). A deep sternal wound infection caused by *Ureaplasma urealyticum*. J Med Microbiol.

[12] Geissdörfer W, Sandner G, John S, Gessner A, Schoerner C, Schröppel K (2008). *Ureaplasma urealyticum* meningitis in an adult patient. J Clin Microbiol.

[13] Pailhoriès H, Chenouard R, Eveillard M (2019). A case of *Ureaplasma parvum* meningitis in an adult after transphenoidal ablation of craniopharyngioma. Int J Infect Dis.

[14] Kanat A (1998). Risk factors for neurosurgical site infections after craniotomy: a prospective multicenter study of 2944 patients. Neurosurgery.

[15] Beeton ML, Spiller OB (2017). Antibiotic resistance among *Ureaplasma* spp. isolates: cause for concern?. J Antimicrob Chemother.

[16] Silwedel C, Speer CP, Haarmann A, Fehrholz M, Claus H, Buttmann M (2018). Novel insights into neuroinflammation: bacterial lipopolysaccharide, tumor necrosis factor α, and *Ureaplasma* species differentially modulate atypical chemokine receptor 3 responses in human brain microvascular endothelial cells. J Neuroinflammation.

[17] Silwedel C, Haarmann A, Fehrholz M, Claus H (2019). More than just inflammation: *Ureaplasma* species induce apoptosis in human brain microvascular endothelial cells. J Neuroinflammation.

[18] Silwedel C, Speer CP, Haarmann A, Fehrholz M, Claus H, Schlegel N (2019). *Ureaplasma* species modulate cell adhesion molecules and growth factors in human brain microvascular endothelial cells. Cytokine.

[19] Wang X, Greenwood-Quaintance KE, Karau MJ (2017). *Ureaplasma parvum* causes hyperammonemia in a pharmacologically immunocompromised murine model. Eur J Clin Microbiol Infect Dis.

[20] Xiao L, Crabb DM, Dai Y (2014). Suppression of antimicrobial peptide expression by *Ureaplasma* species. Infect Immun.

[21] Gu W, Deng X (2021). Rapid pathogen detection by metagenomic next-generation sequencing of infected body fluids. Nat Med.

[22] Palacios G, Druce J, Du L, Tran T, Birch C, Briese T, Conlan S, Quan PL, Hui J, Marshall J (2008). A new arenavirus in a cluster of fatal transplant-associated diseases. N Engl J Med.

[23] Han D, Li Z, Li R (2019). mNGS in clinical microbiology laboratories: on the road to maturity. Crit Rev Microbiol.

[24] Miao Q, Ma Y, Wang Q, Pan J, Zhang Y, Jin W, Yao Y, Su Y, Huang Y, Wang M (2018). Microbiological diagnostic performance of metagenomic next-generation sequencing when applied to clinical practice. Clin Infect Dis.

[25] Parize P, Muth E, Richaud C, Gratigny M, Pilmis B, Lamamy A, Mainardi JL, Cheval J, de Visser L, Jagorel F (2017). Untargeted next-generation sequencing-based first-line diagnosis of infection in immunocompromised adults: a multicentre, blinded, prospective study. Clin Microbiol Infect.

[26] Charalampous T, Kay GL, Richardson H, Aydin A, Baldan R, Jeanes C, Rae D, Grundy S, Turner DJ, Wain J (2019). Nanopore metagenomics enables rapid clinical diagnosis of bacterial lower respiratory infection. Nat Biotechnol.

[27] Wilson MR, Shanbhag NM, Reid MJ, Singhal NS, Gelfand JM, Sample HA, Benkli B, O’Donovan BD, Ali IKM, Keating MK (2015). Diagnosing balamuthia mandrillaris encephalitis with metagenomic deep sequencing. Ann Neurol.

[28] Deetjen P, Maurer C, Rank A, Berlis A, Schubert S, Hoffmann R (2014). Brain abscess caused by *Ureaplasma urealyticum* in an adult patient. J Clin Microbiol.

[29] Pigrau C, Almirante B, Gasser I, Pahissa A (1995). Sternotomy infection due to *Mycoplasma hominis* and *Ureaplasma urealyticum*. Eur J Clin Microbiol Infect Dis.

